# Lidocaine Affects Collagen Breakdown Without Compromising Cell Viability in Cultured Human Tenocytes: An In Vitro Study

**DOI:** 10.3390/cells14130988

**Published:** 2025-06-27

**Authors:** Filippo Randelli, Manuel G. Mazzoleni, Alessandra Menon, Alberto Fioruzzi, Dolaji Henin, Michele Sommariva, Nicoletta Gagliano

**Affiliations:** 1 Hip Department (CAD), Gaetano Pini-CTO Orthopedic Institute, University of Milan, P-za Cardinal Ferrari 1, 20122 Milano, Italy; filippo.randelli71@gmail.com (F.R.); manuelmazzoleni4@gmail.com (M.G.M.); alberto.fioruzzi@gmail.com (A.F.); 2 Laboratory of Applied Biomechanics, Department of Biomedical Sciences for Health, Università Degli Studi di Milano, Via Mangiagalli 31, 20133 Milan, Italy; ale.menon@me.com; 3 U.O.C. 1° Clinica Ortopedica, Gaetano Pini-CTO Orthopedic Institute, University of Milan, P-za Cardinal Ferrari 1, 20122 Milano, Italy; 4 Department of Biomedical, Surgical and Dental Sciences, Università Degli Studi di Milano, Via Mangiagalli 31, 20133 Milan, Italy; dolaji.henin@unimi.it; 5 Department of Biomedical Sciences for Health, Università degli Studi di Milano, Via Mangiagalli 31, 20133 Milan, Italy; michele.sommariva@unimi.it

**Keywords:** lidocaine injection, tendinopathy, infiltration, tendon, tenocytes, apoptosis, collagen turnover

## Abstract

Local anesthetics (LAs) are frequently administered via peritendinous ultrasound-guided injections for diagnostic and therapeutic purposes. Since in vitro studies have demonstrated LAs’ tenotoxic effects, raising concerns about their safety in infiltrative treatments, and since lidocaine (LD) emerged as one of the most cytotoxic LAs, we analyzed apoptosis, oxidative stress, and collagen turnover pathways in human tenocytes treated with LD, as well as the possible protection from LD-induced injury elicited by antioxidant ascorbic acid (AA). Tenocytes from gluteal tendons were treated with 0.2 and 1 mg/mL LD, or left untreated (CT), and treated with 50 μg/mL or 250 μg/mL AA. Nuclear morphology, cytochrome c expression, and caspase 3 activation were analyzed to study the effect of LD on apoptosis. Heme Oxygenase 1 (*HO-1*) mRNA and genes and proteins involved in collagen turnover were investigated using molecular approaches. Our results show that 0.2 and 1 mg/mL LD did not induce apoptosis and did not modify collagen synthesis and maturation. Conversely, increased collagen degradation was observed, and AA was not protective against oxidative stress induction in the presence of LD. Our findings suggest that LD does not affect the cell viability of tenocytes and that peritendinous LD injections are safe in this regard. LD-associated collagen degradation and the AA buffer effect are still debatable. Overall, our study contributes to clarifying the effect of LD on tenocytes’ viability and ECM homeostasis and provides new additional information useful for the safe clinical application of this drug and for further analysis.

## 1. Introduction

Tendinopathies are degenerative pathological conditions characterized by histological alterations, including disorganization of collagen fibrils, an increased relative proportion of proteoglycans, glycosaminoglycans, and non-collagenous components of the extracellular matrix (ECM), accompanied by neovascularization and inflammatory processes [1,2]. These structural and biochemical changes determined by ECM alteration and turnover imbalance result in painful symptoms often associated with significant biomechanical functional impairments and disability.

Greater trochanteric pain syndrome (GTPS), a condition also referred to as lateral hip pain syndrome, is a multifaceted clinical entity characterized by chronic and recurrent pain in the lateral hip region and localized to the greater trochanter of the femur. This syndrome arises from a combination of biomechanical dysfunctions and anatomo-histological changes affecting the peritrochanteric structures. Recent investigations into GTPS have identified that in most cases, the syndrome is predominantly caused by degenerative tendinopathy of the gluteus medius and minimus muscles, resulting in a debilitating pain syndrome [3,4].

Despite its high prevalence, the management of GTPS, as with tendinopathies more broadly, remains a significant clinical challenge [3,5].

The cellular pathophysiological mechanisms and biomechanical etiopathogenetic factors underlying these conditions are still incompletely understood, and many therapeutic interventions rely on empirical approaches [6,7,8,9,10].

In cases where conservative treatments, such as oral anti-inflammatory therapies and physiotherapy, fail, or when differential diagnosis proves difficult, infiltrative therapies using corticosteroids and LA are employed to achieve rapid pain relief. These infiltrative procedures are reported to provide pain alleviation in 60–100% of patients [4].

LAs are frequently utilized in combination with corticosteroids for diagnostic and therapeutic purposes, particularly to manage pain and facilitate functional recovery in young athletes and individuals affected by trauma or repetitive microtrauma [11]. Commonly, these agents are locally administered via peritendinous ultrasound-guided injections. Peritrochanteric infiltrations with LD, either alone or in combination with other agents, provide notable clinical advantages, including immediate pain relief, reduced procedural discomfort, and precise localization of the pain source for diagnostic purposes [12]. Moreover, LD can also act as a potent anti-inflammatory agent and its anti-inflammatory properties were studied and compared with those of steroids and non-steroidal drugs (NSAIDs) [13].

Nevertheless, in vitro studies have demonstrated the cytotoxic effects of LA, including LD, on tenocytes, raising concerns about their safety in infiltrative treatments. While the degenerative risks associated with corticosteroid infiltrations are well documented [11], the impact of LAs on tendons, particularly on the tendons of the gluteus medius and minimus, requires further investigation to ensure their safe application in peritrochanteric therapy.

Thus, more recently, attention has shifted toward the cytotoxic potential of LAs on cellular populations targeted by infiltrative therapeutic protocols [14,15,16,17,18,19,20,21]. It was reported that LA may cause cell toxicity in different cell types [14,15,16,17], including tenocytes [22,23,24,25,26,27,28].

To understand how LAs may cause cell and tissue damage and to provide a deeper knowledge of the mechanisms of local anesthetic-induced cytotoxicity, most studies have focused on characterizing the effect of local anesthetics on cell viability and apoptosis, and their results suggested that LAs can exert a cytotoxic effect triggering cell apoptosis and promoting the intracellular generation of reactive oxygen species [25].

Apoptosis, induced by both exogenous and endogenous stimuli that trigger two pathways, the intrinsic and the extrinsic [29,30], is an important component in maintaining homeostasis in many adult tissues [31]. In tendons, it was suggested to act as a primary cause of tendinopathy and tears, since it severely contributes to the loss of tendon homeostasis [32]. Because the concentration of LD required to treat cells was not clearly defined in previous studies describing the effect of LD on tenocytes, in this study, we aimed to investigate the effect of LD on apoptosis in cultured tenocytes.

Oxidative stress, caused by an imbalance between reactive oxygen species (ROS) and antioxidants, is a crucial player involved in various musculoskeletal disorders [33]. Also, intracellular ROS production was significantly increased upon LA treatment in tenocytes [25].

To mitigate the oxidative stress-induced cytotoxic effects of LAs, various strategies have been explored in vitro. Antioxidants such as cyanidin, N-acetylcysteine, and ascorbic acid (vitamin C) (AA) have demonstrated significant efficacy in enhancing the survival of tenocytes and chondrocytes exposed to LA. Indeed, ascorbic AA, a well-characterized antioxidant that can prevent the formation of free radicals [34,35,36], was reported as protecting hamstring-derived tenocytes from oxidative stress [35].

Tenocytes are the main actors in maintaining tendon homeostasis. Indeed, tendon biomechanical properties are based on the integrity of their extracellular matrix (ECM), mainly consisting of type I collagen (COL-I) [37,38], which accounts for the 60–85% of the dry mass of the tendon and about 95% of the total collagen.

Collagen is therefore the major component of the tendon extracellular matrix (ECM); its content is tuned by a finely regulated dynamic turnover carried out by tenocytes that are specialized fibroblasts interspersed between collagen fibers and responsible for collagen synthesis, maturation, and degradation. Indeed, tenocytes orchestrate the mechanisms responsible for tendon ECM homeostasis that is crucial in the ability to resist tensile forces, to adapt to mechanical loading, and to repair in response to injury [37,38].

Since ECM is crucial in the tendon homeostasis, as well as in the healing process, and since previous studies did not analyze the eventual effect of LA on the overall tenocyte activity in ECM remodeling, we aimed at characterizing the cytotoxic effects of LD on human tenocytes in vitro and the eventual protective effects of AA in relation to the mechanisms involved in maintaining collagen and ECM balance. For this purpose, collagen turnover pathways, as well as apoptosis and oxidative stress, were investigated using morphological and molecular approaches. The results of this study could provide new insights into the context of advancing therapeutic strategies for GTPS and, more generally, for tendinopathies, in both diagnostic and therapeutic infiltrative procedures.

## 2. Materials and Methods

### 2.1. Samples

Fragments from human gluteal tendon were obtained from 6 patients (mean age 52.67 ± 7.66 years; 4 males and 2 females) undergoing total hip replacement through an anterior approach, without any gluteal tendon pathology. For each sample, the mid-substance of the collected tendon, possessing the typical structure of the dense regular connective tissue of this organ, was isolated and analyzed.

All subjects provided their informed consent to be included in the study. The study was conducted in accordance with the Declaration of Helsinki, and the protocol was approved by the Local Ethics Committee (Comitato Etico Milano Area 2) (code of the protocol: LIDO-VITC, approval date 28 July 2022).

### 2.2. Primary Cell Cultures

Tendon fragments were immediately washed in sterile PBS, plated in T25 flasks in Dulbecco’s Modified Eagle Medium (DMEM) (Euroclone, Pero, Milan, Italy) supplemented with 10% heat-inactivated fetal bovine serum (FBS) (Life Technologies, Segrate, Milan, Italy), antibiotics (100 U/mL penicillin, 0.1 mg/mL streptomycin) (Euroclone, Pero, Milan, Italy), and incubated at 37 °C in a humidified atmosphere containing 5% CO_2_. When tenocytes grew out from the explant, they were trypsinized (0.025% trypsin-0.02% EDTA) for secondary cultures and plated in T75 flasks. Cell viability was assessed using the trypan blue exclusion method. Confluent human tenocytes were used between the fourth and fifth passage for the analysis. Cells were cultured in serum-free DMEM to obtain the serum-free cell supernatants for slot blot and SDS-zymography analysis. To analyze the effect of LD, tenocytes were treated with 0.2 and 1 mg/mL LD diluted in the cell culture medium, in absence (LD) or in presence of ascorbic (LD + AA) acid at different concentrations. To maintain collagen synthesis and maturation, AA was used at the 50 μg/mL concentration (AA50) [39,40] while, to obtain an antioxidant effect, it was used at the 250 μg/mL concentration (AA250). Untreated cells served as controls (CTs).

Tenocytes derived from each patient and cell supernatants were prepared in duplicate and analyzed after 48 h.

### 2.3. Real-Time PCR

Total RNA (1 µg) was reverse transcribed in 20 µL final volume of reaction mix (Biorad, Segrate, Milan, Italy). The expression of genes involved in collagen turnover pathways was analyzed by real-time RT-PCR in samples run in triplicate. GAPDH mRNA levels were used for the normalization of the expression of the target genes in each sample. The sequences of the primers used in this study were the following: glyceraldehyde 3-phosphate dehydrogenase (GAPDH): forward CCCTTCATTGACCTCAACTACATG, reverse TGGGATTTCCATTGATGACAAGC; collagen type I (COL-I): forward CGACCTGGTGAGAGAGGAGTTG; reverse AATCCATCCAGACCATTGTGTCC; long lysyl hydroxylase 2 (LH2b): forward CCGGAAACATTCCAAATGCTCAG, reverse GCCAGAGGTCATTGTTATAATGGG; lysyl oxidase (LOX): forward GGATACGGCACTGGCTACTT; reverse GACGCCTGGATGTAGTAGGG; tissue inhibitor of matrix metalloproteinase 1 (TIMP-1): forward GGCTTCTGGCATCCTGTTGTTG, reverse AAGGTGGTCTGGTTGACTTCTGG; tissue inhibitor of matrix metalloproteinase 2 (TIMP-2): forward TGGAAACGACATTTATGGCAACCC, reverse CTCCAACGTCCAGCGAGACC. Gene expression was assayed in triplicate samples in a Bioer LineGene 9600 thermal cycler (Bioer, Hangzhou, China) after 40 cycles. The cycle threshold (Ct) was determined, and gene expression levels relative to that of GAPDH were calculated.

### 2.4. Slot Blot

Collagen type I (COL-I) and matrix metalloproteinase (MMP)-1 protein levels secreted by primary tenocytes were assessed in serum-free cell supernatants by slot blot as previously described [41]. Briefly, serum-free supernatants (100 μg of total proteins) from untreated and LD-treated cells in the different experimental groups were spotted on a nitrocellulose membrane in a BioDot SF apparatus (Bio Rad, Segrate, Milan, Italy). Membranes were blocked for 10 min in EveryBlot Blocking Buffer (Bio Rad, Segrate, Milan, Italy) and then incubated for 1 h at room temperature with primary polyclonal antibodies anti-collagen I (1:1000 Abcam ab34710) (Prodotti Gianni, Milan, Italy) or anti-MMP-1 (1:1000, Invitrogen PA5-27210) (Fisher Scientific, Segrate, Milan, Italy) in 1X Tris-buffered saline containing 0.05% Tween 20 (TBST) (Sigma-Aldrich, Milan, Italy). After 1 h of incubation with a secondary anti-rabbit HRP-conjugated antibody (1:20,000 in TBST; Sigma A8725) (Sigma-Aldrich, Milan, Italy), membranes were incubated using the Amplified Opti-4CN substrate (Amplified Opti-4CN, Bio Rad, Segrate, Milan, Italy) to reveal the immunoreactive bands that were quantified by densitometric scanning (UVBand, Eppendorf, Milan, Italy).

### 2.5. SDS-Zymography

Sodium dodecyl sulphate polyacrylamide gel electrophoresis (SDS-PAGE) using 10% polyacrylamide gels co-polymerized with 1 mg/mL type I gelatine was used to obtain the zymograms of serum-free cell culture supernatants (10 μg of total proteins per sample). After the run at 4 °C, the gels were washed twice in 2.5% Triton X-100 for 30 min each, incubated overnight in a substrate buffer at 37 °C (50 mM Tris-HCl, 5 mM CaCl_2_, 0.02% NaN_3_, pH 7.5), and stained using Coomassie blue. MMP-2 gelatinolytic activity was detected after destaining the gels as clear bands on a blue background; these were quantified by densitometric scanning (UVBand, Eppendorf, Milan, Italy).

### 2.6. Hoechst Staining

The presence of apoptosis in LD-treated cells was analyzed using Hoechst 33342 (bisBenzimide H33342); this allows one to detect the compacted state of chromatin in apoptotic cells using a fluorescence microscope [42]. The DNA in the condensed chromatin of apoptotic cells is more brightly stained than the chromatin of normal cells upon incubation with the blue-fluorescent Hoechst 33342 dye. Briefly, tenocytes (2 × 10^4^) were seeded and cultured on 12 mm diameter round coverslips in 24-well multiwell plates. When cells attached, fresh medium containing LD and AA at the established concentration was added. Cells cultured in DMEM served as CT. After 48 h, cells were fixed in 4% paraformaldehyde in PBS containing 2% sucrose for 5 min at room temperature. After fixation, cells were postfixed in 70% ethanol and stored at −20 °C until use. To detect apoptotic tenocytes, cells were incubated with 1 μg/mL Hoechst 33342 in PBS for 20 min. Slides were mounted onto glass slides using Mowiol (Sigma-Aldrich) mounting medium and observed using a fluorescence microscope (Nikon Eclipse 80i) (Nikon, Amstelveen, The Netherlands).

### 2.7. Immunocytochemistry

Tenocytes were seeded and cultured on 12 mm diameter round coverslips placed in 24-well multiwell plates, as described for the Hoechst staining. After washing with PBS, cells were incubated for 1 h at room temperature with the monoclonal primary antibody anti-cytochrome c (1:100; sc-13156, Santa Cruz Biotechnology, Santa Cruz, CA, USA) and, after washing, with the secondary antibody conjugated with Alexa 488 (1:500; Molecular Probes, Invitrogen, Segrate, Milan, Italy) for 1 h at room temperature. Finally, the coverslips were stained for 10 min with 4′,6-diamidino-2-phenylindole (DAPI) and mounted onto glass slides using Mowiol (Sigma-Aldrich, Milan, Italy) mounting medium. Cells were observed by a WD THUNDER Imager Tissue 3D (Leica Microsystems, Buccinasco, Milan, Italy).

### 2.8. 2′,7′-Dichlorodihydrofluorescein Diacetate (DCFDA) Assay

DCFDA (MedChem, Monmouth Junction, NJ 08852, USA) was diluted in DMSO at the concentration of 10 mM, and the working concentration to treat the cells was 10 µM in DMEM. The assay was performed on tenocytes seeded and cultured on 12 mm diameter round coverslips placed in 24-well multiwell plates. After washing with PBS, the coverslips were incubated for 45 min at 37 °C in the dark, washed in PBS, and incubated with 1 μg/mL Hoechst 33342 in PBS for 10 min. Tenocytes treated with 100 µM of H_2_O_2_ to induce oxidative stress served as positive controls. Finally, slides were mounted onto glass slides using Mowiol (Sigma-Aldrich) mounting medium and observed using the fluorescence microscope (Nikon Eclipse 80i).

### 2.9. Western Blot Analysis

Western blot analysis was used to analyze caspase-3 expression in tenocytes’ whole-cell lysates. Cell lysates (20 μg of total proteins) were diluted in sample buffer (Bio-Rad Laboratories, Segrate, Italy) and run using SDS-PAGE under reducing and denaturing conditions. The gels were transferred onto nitrocellulose membranes. Membranes were incubated with a polyclonal anti-caspase-3 antibody that detects endogenous levels of full-length caspase-3 (35 kDa) and the large fragment of caspase-3 resulting from cleavage (17 kDa) (Cell Signaling 9662, Leiden, The Netherlands). Tenocytes treated with 40 µM of staurosporine (Sigma-Aldrich, Milan, Italy) for 4 h at 37 °C to induce apoptosis served as positive controls. For normalization, membranes were reprobed with a monoclonal α-tubulin antibody (1:2000, Sigma-Aldrich, Milan, Italy).

### 2.10. Statistical Analysis

Statistical analysis was performed using the Prism v 9.3 software (GraphPad Software, San Diego, CA, USA). Data were expressed as mean ± standard deviation (SD). One-way ANOVA was used to compare the experimental groups. Differences associated with *p*-values lower than 5% were considered significant.

## 3. Results

### 3.1. Apoptosis

**Figure 1:**
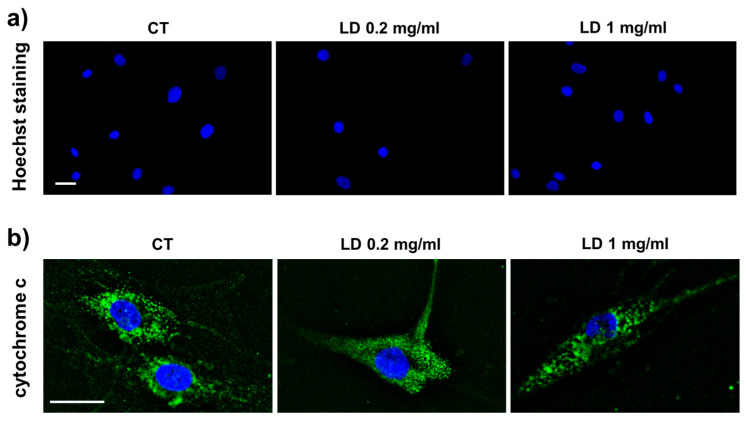
(**a**) Micrographs of Hoechst 33342-stained tenocytes observed using a fluorescence microscope showing that LD-treated cells do not exhibit, after 48 h, the nuclear condensation observed in apoptotic cells and revealing that their nuclear morphology is similar to CT. (**b**) Micrographs showing immunofluorescence detection of cytochrome c in CT and LD-treated tenocytes. CT cells showed a punctate cytoplasmic staining pattern that is typical for mitochondrial localization. A similar pattern was observed 48 h after LD administration. Green: cytochrome c; blue: DAPI. Scale bar: (**a**) 20 µm; (**b**) 25 µm.

To assess whether LD can induce apoptosis, analysis was performed on cells cultured in the absence of AA. Fluorescence microscopy of Hoechst-stained cells revealed that both control (CT) and LD-treated cells had normal nonapoptotic nuclei (Figure 1a). In line with this, cytochrome c immunocytochemistry showed that both CT and LD-treated samples had similar cytochrome c punctate cytoplasmic immunoreactivity that is typical of nonapoptotic cells when the molecule is retained in non-damaged mitochondria, suggesting that mitochondrial integrity is not affected by the drug (Figure 1b).

**Figure 2:**
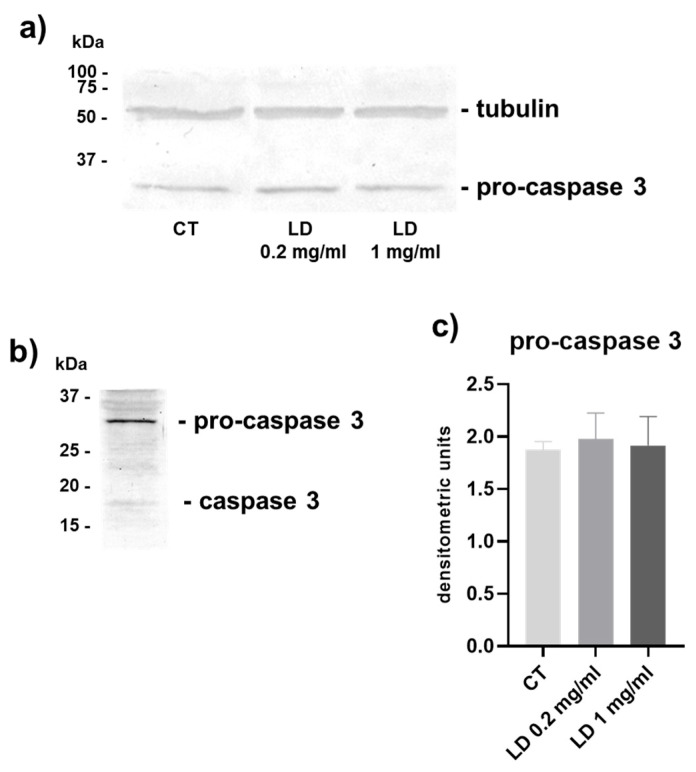
(**a**) Representative Western blot analysis of caspase-3 expression showing the full-length protein detectable in CT and cells treated with LD for 48 h. (**b**) Representative Western blot of tenocytes treated with staurosporine to induce apoptosis used as a positive control, as described in the Materials and Methods section. (**c**) Bar graphs showing pro-caspase-3 levels after densitometric scanning of immunoreactive bands obtained in at least 3 independent experiments. Data are means ± SD.

Furthermore, the level of pro-caspase 3, evaluated by Western blot and densitometric analysis, was superimposable in the two experimental groups, whilst activated caspase 3 remained undetectable, corroborating the suggestion that LD does not induce apoptotic cell death in tenocytes (Figure 2a–c).

### 3.2. Oxidative Stress

**Figure 3:**
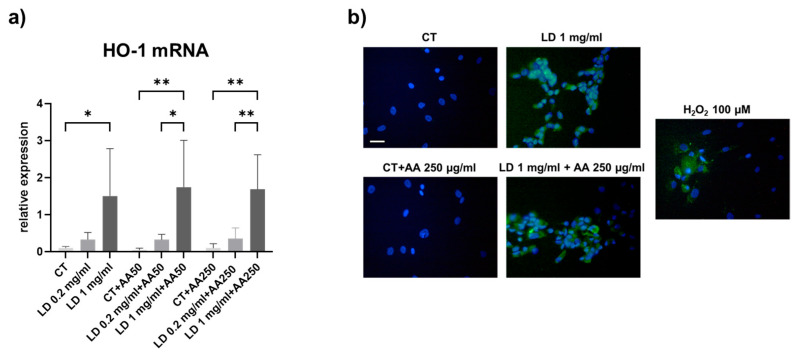
(**a**) Representative bar graphs showing *HO-1* gene expression in tenocytes treated with 0.2 and 1 mg/mL LD and different doses of AA (50 and 250 μg/mL) for 48 h, as described in the Materials and Methods section. Data were obtained in at least 2 independent amplifications with samples run in triplicate and are means ± SD. * *p* < 0.05; ** *p* < 0.01. (**b**) Micrographs of tenocytes after DCFDA incubation showing the presence of ROS in LD-treated cells. Nuclei were stained with Hoechst. Scale bar: 20 µm.

To investigate the ability of LD to trigger oxidative stress in LD-treated tenocytes, the gene expression of HO-1 was analyzed. Our results show a dose-dependent increase in HO-1 mRNA levels in tenocytes treated with LD (Figure 3a). In the absence of AA, HO-1 was progressively upregulated upon increasing LD concentration (*p* = 0.05 and *p* < 0.05 for 0.2 and 1 mg/mL LD, respectively, compared to the matched CT). The pattern of HO-1 was not affected by the increasing doses of AA utilized to counteract the possible oxidative stress. Indeed, LD significantly increased HO-1 mRNA levels in the presence of both 50 and 250 μg/mL of AA (*p* < 0.05 and *p* < 0.01 for 1 mg/mL LD + AA50 vs. 0.2 mg/mL LD + AA50 and CT, respectively; *p* < 0.01 for 1 mg/mL LD + AA250 vs. 0.2 mg/mL LD + AA250 and CT, respectively).

To evaluate the presence of oxidative stress in tenocytes upon LD treatment, we performed a DCFDA assay. Analysis using the fluorescence microscope showed that 0.2 and 1 mg/mL LD induced oxidative stress. Moreover, as observed for HO-1 gene expression, AA was not able to revert ROS content to the CT levels (Figure 3b).

### 3.3. Collagen Turnover Pathways: Synthesis and Maturation

**Figure 4:**
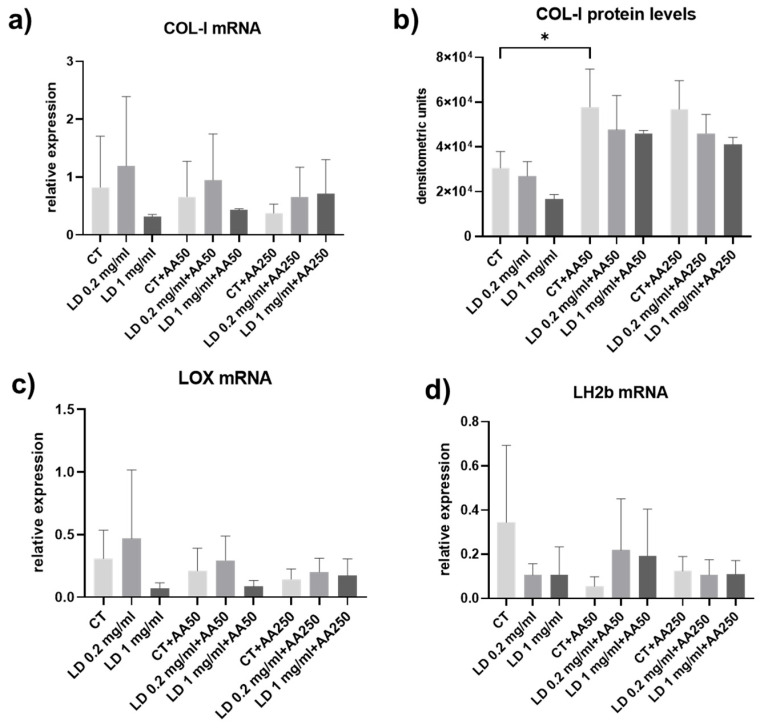
(**a**) Bar graphs showing *COL-I* mRNA levels, (**b**) COL-I protein levels secreted in the cell culture supernatants, and (**c**) *LOX* and (**d**) *LH2b* gene expression in cells harvested after 48 h. Data showing mRNA levels were obtained in at least 2 independent amplifications with samples run in triplicate and are means ± SD. * *p* < 0.05; *p* = 0.06 for CT + AA250 vs. CT.

Since COL-I is the main collagen in tendon ECM, its expression was analyzed both at the mRNA and protein level. *COL-I* gene expression was not significantly affected in the experimental groups considered, although it tended to be lower after administration of 1 mg/mL LD in the absence of a protective dose of AA (Figure 4a). Also, COL-I protein levels secreted in the cell supernatants were not significantly influenced by LD administration. However, a trend towards a decrease in COL-I released by tenocytes can be observed in cells cultured in the absence of AA and using increasing doses of LD (Figure 4b). Tenocytes cultured in the absence of AA had the lowest levels of COL-I expression, confirming the importance of vitamin C as a co-factor needed for the COL synthesis process.

Collagen maturation was studied by analyzing the gene expression of *LH2b* and *LOX*, key enzymes involved in hydroxylation of specific amino acids and in the formation of covalent cross-links upon secretion of newly synthetized collagen in the extracellular environment [43,44]. Indeed, although cross-linking occurs extracellularly after collagen secretion by LOX, the cross-links depend on the previous intra-cellular post-translational modifications to the collagen molecule by LH in the rough endoplasmic reticulum, leading to the hydroxylation of lysine residues. Our results show that *LH2b* and *LOX* gene expression was not influenced by LD and AA in the experimental conditions considered (Figure 4c,d).

### 3.4. Collagen Turnover Pathways: Degradation

Collagen degradation pathways were assayed by analyzing MMP-1 levels and -2 activity in cell culture supernatants and the gene expression for their inhibitors, *TIMP-1* and 2.

**Figure 5:**
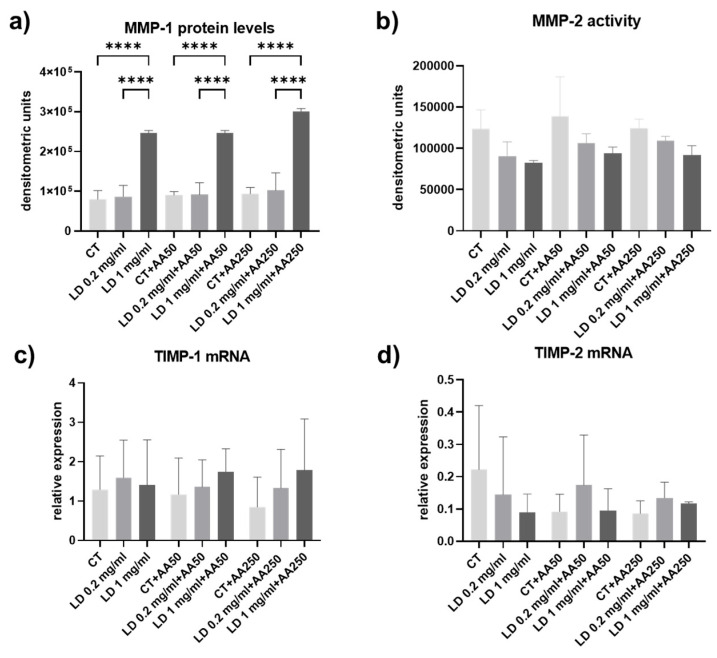
Bar graphs showing (**a**) MMP-1 and (**b**) MMP-2 protein levels secreted in the cell culture supernatants, (**c**) *TIMP-1* and (**d**) *TIMP-2* gene expression in cells analyzed after 48 h. Data in (**a**,**b**) were obtained in at least 2 independent experiments; data in (**c**,**d**) were obtained in at least 2 independent amplifications with samples run in triplicate. Data are means ± SD. **** *p* < 0.0001.

MMP-1 levels were assayed in the cell culture supernatants using slot blot. Densitometric analysis of immunoreactive bands showed a similar pattern of expression in the absence of AA and after administration of AA. However, significant upregulation was induced by LD (Figure 5a). Indeed, the administration of 1 mg/mL LD increased MMP-1 secretion compared to the matched CT (*p* < 0.0001 for 1 mg/mL LD and 1 mg/mL LD + AA250 and *p* < 0.001 for 1 mg/mL LD + AA50 samples treated with CT). A significant upregulation of MMP-1 levels in cell culture supernatants was also detected in samples treated with 1 mg/mL compared to matched 0.2 mg/mL LD (*p* < 0.0001 for 1 mg/mL LD and 1 mg/mL LD + AA250 and *p* < 0.001 for 1 mg/mL LD + AA50). MMP-2 levels and activity were not influenced by LD or AA administration (Figure 5b). Also, *TIMP-1* and -*2* mRNA levels were not influenced by LD or AA administration (Figure 5c,d).

## 4. Discussion

LAs have been demonstrated to cause cell toxicity to many different cell types [14,15,16,17], including tenocytes [22,23,24,25,26] in a time- and dose-dependent manner. LD emerged as one of the most cytotoxic LAs able to induce apoptosis- and necrosis-associated morphological changes, as well as the production of ROS in treated tenocytes [25,27,28]. Since most studies have focused on apoptosis and oxidative stress, while tenocyte’s ability to maintain the ECM homeostasis and turnover has only been partially investigated, in our study, we were interested in characterizing the overall effect of LD in cultured human tenocytes to dissect the overall collagen turnover pathways. Indeed, the expression of COL-I and III was described [45], while the effect of LD on collagen maturation, as well as interstitial collagen degradation, remain still largely unexplored.

For this purpose, we analyzed, for the first time, to our knowledge, collagen turnover pathways, including maturation, secretion, and degradation in human tenocytes after treatment with different doses of LD. In our experimental setting, apoptosis and oxidative stress were also investigated.

Different concentrations of AA are essential in tenocyte cultures to enhance collagen synthesis [39,40]. AA not only promotes collagen biosynthesis but is also a well-characterized antioxidant that can prevent the formation of free radicals [36]. It was previously reported that AA can protect hamstring-derived tenocytes from oxidative stress [35], and the effect of different concentrations of AA on cultured human tenocytes was characterized in real-time, showing that a concentration of AA ranging from 10 to 250 μg/mL did not significantly influence cell viability after 24 h and up to 148 h [46]. In our study, AA was used at a 50 μg/mL concentration (AA50) to maintain collagen synthesis and at a 250 μg/mL (AA250) to obtain an antioxidant effect. Interestingly, the expected antioxidant effect of AA was not detected in our study at this concentration.

Apoptosis has an important role in maintaining homeostasis in many adult tissues [31], and in tendons, apoptosis was suggested as a primary cause of tendinopathy and tears [32]. Apoptosis can be induced by both exogenous and endogenous stimuli that trigger two pathways, the intrinsic and the extrinsic; both pathways converge on the activation of caspases that act as the main executors of the events leading to cell death [29,30]. The intrinsic pathway involves mitochondria, leading to an increase in mitochondrial membrane permeability. This results in the release of normally sequestered proapoptotic proteins such as cytochrome c that can induce the activation of caspase-9 which, finally, activates other caspases such as caspase-3 and -7 [29,30]. The extrinsic pathway is activated by transmembrane receptors which are members of the tumor necrosis factor gene, inducing apoptosis by activating caspase 8.

The increase in apoptotic tenocytes could be detrimental in tendons and responsible for changes such as loss of fiber structure and arrangement, collagen turnover imbalance, and reduced ability to heal after injury, thus severely contributing to the loss of tendon homeostasis. Previous studies described the effect of LAs, including LD, on tenocytes, and reported both apoptotic and necrotic effects [25]. The very evident cytotoxic effect was dose-dependent. In these studies, however, the working concentration of LD was not clearly defined, since most of them refer to the 1% or 2% LD likely intended as the clinical doses for injection, that are, respectively, 10 mg/mL and 20 mg/mL and, therefore, very high and possibly cytotoxic for in vitro analysis [27].

Our findings do not confirm this apoptotic effect and strongly suggest that 0.2 and 1 mg/mL LD used in our experimental conditions did not induce apoptosis in cultured tenocytes. The discrepancy could be therefore based on the dose of LD used to treat the cells.

Indeed, in our experimental setting, the absence of apoptosis was clearly demonstrated using very different technical approaches upon LD administration, based on the analysis of nuclear morphology, the pattern of expression of cytochrome c (which, in physiological conditions, has mitochondrial localization), and the expression of caspase 3 in whole-cell lysates.

Interestingly, it was recently demonstrated that LD significantly inhibits tenocytes from entering the S-phase of the cell cycle, suggesting that LD causes cell cycle arrest by preventing tenocytes from advancing from the G1 phase to the S phase. Moreover, this finding was also supported by the observation that LD dose-dependently downregulated the expression of cyclin A and CDK2 and increased p21, p27, and p53, thus blocking the progression of treated tenocytes through the cell cycle. By contrast, there was no statistically significant evidence of apoptosis. Our results are in line with these findings that were obtained using a similar concentration of LD [45].

Oxidative stress has been implicated in governing normal physiological activities and pathological processes. An excess formation of reactive oxygen species (ROS) can be determined by an imbalance between ROS and antioxidants, leading to damage to cellular components, such as nucleic acids, proteins, and lipids, therefore affecting cellular and tissue homeostasis. In the musculoskeletal system, ROS have been implicated in several disorders, including rheumatoid arthritis, osteoporosis, and tendinopathy [47,48]. Indeed, the administration of antioxidants such as vitamin C, curcumin, nicotinamide mononucleotide, and superoxide dismutase for the therapy of tendinopathy was demonstrated to constitute a beneficial treatment option for tendon disorders in in vitro and in vivo studies [49,50,51,52,53].

It was previously demonstrated that LAs, including LD, lead to the generation of ROS as determined by confocal microscopy after incubation with 2′,7′-dichlorofluorescin diacetate (DCFDA) and by fluorescence-activated cell sorting analysis [25].

In our study, we assayed the stimulation of oxidative stress in tenocytes after LD treatment by analyzing the expression of HO-1, which is undetectable under basal conditions but highly inducible under conditions of stress or inflammation, representing a well-recognized indicator of oxidative stress under pro-oxidative conditions [54]. Indeed, three HO isoforms (HO-1, HO-2, HO-3) have been described. While HO-2 is constitutively expressed, and HO-3 is only found in rats, HO-1 is normally expressed at low levels in most tissues but is highly inducible by a variety of stimuli [55,56].

Our results show that HO-1 gene expression is dose-dependently induced by LD and that AA administration was not able to restore the basal expression detected in CT. Moreover, HO-1 was upregulated at a similar extent in the absence of AA or upon treatment with 50 or 250 μg/mL, likely suggesting that AA is not effective in protecting from LD-induced oxidative stress, as confirmed also after DCFDA incubation.

Type I collagen (COL-I) is the major component of tendon ECM; its content is tuned by a finely regulated dynamic turnover carried out by tenocytes that are responsible for collagen synthesis, maturation, and degradation, thus determining the tendon ECM homeostasis and the ability to resist tensile forces, adapt to mechanical loading, and repair in response to injury [37,38]. We investigated *COL-I* gene and protein levels in cell culture supernatants, and our data suggest that *COL-I* mRNA was not affected by LD and AA administration. COL-I protein levels were detected at higher degrees, as expected, in cells treated with AA, confirming its role as a cofactor in maintaining optimal collagen synthesis. However, no significant effects were elicited by LD, suggesting that the step of collagen secretion by tenocytes in the extracellular environment is preserved in the presence of LD.

During maturation, newly synthesized collagen undergoes cross-linking, a key step needed to provide collagen fibril stabilization and tendon tensile strength following the hydroxylation of amino acid residues by lysyl oxidase (*LOX*) and lysyl hydroxylase (*LH*) [43,44]. We investigated both *LOX* and *LH2b* gene expressions to evaluate whether LD could have an impact on collagen maturation. Our data show that both LOX and LH2b, the LH isoform usually involved in deranged ECM remodeling, remained unaffected, suggesting that collagen maturation is not targeted by LD treatment.

Since collagen content is the result of a finely regulated and dynamic balance between its synthesis and degradation driven by MMPs, tendon strength is strongly dependent on degradation pathways. COL breakdown is performed by MMP-1, which exerts its collagenolytic activity to drive the intact collagen triple helix, allowing further degradation by other proteases such as MMP-2 and -9 [57,58,59]. The role of MMP-1 in tendons is crucial, since an inverse correlation between MMP-1 gene/protein expression and the amplitude of mechanical load on tendons occurs, therefore suggesting that low levels of MMP-1 could be consistent with a stronger tendon structure [60]. However, to consider effective collagen degradation, the expression of TIMPs also needs to be investigated. Indeed, TIMPs bind to MMPs to regulate both activation and activity, and TIMP-1 is the main inhibitor of MMP-1 [59].

Our results show that MMP-2 and -9 levels remained unchanged after administration of LD, as well as in the presence of different doses of AA. However, interestingly, MMP-1 protein levels in cell culture supernatants were significantly increased by LD. Although MMP-1 protein levels and not its activity were assayed, this is an important finding, since to the best of our knowledge, the current study is the first to document how LD affects the expression of MMPs in tenocytes, particularly of MMP-1, which plays a crucial role in tendon collagen remodeling.

In relation to this, an increase in ROS species in tendon injury can be responsible for a state of oxidative stress that, in turn, can lead to pro-inflammatory cytokines’ upregulation and extracellular matrix (ECM) degradation [61]. Moreover, the relationship between oxidative stress and collagen degradation was demonstrated in human tendon cells treated with H_2_O_2_ that upregulated MMP-1 at the mRNA and protein level, thus confirming the role of oxidative stress in tendon ECM balance [62].

Our results show that MMP-1 protein levels were significantly upregulated in tenocytes’ supernatants after the administration of 1 mg/mL LD compared to the controls. Moreover, the pattern of expression was similar in the absence of AA or upon treatment with 50 or 250 μg/mL, suggesting that AA is not effective in preventing excessive collagen degradation in LD-treated tenocytes. Interestingly, we observed a similar pattern for *HO-1* gene expression, leading to the hypothesis that oxidative stress and increased collagen breakdown are related and concomitant detrimental effects induced by LD on tenocytes. Although AA was not effective in preventing MMP-1 and *HO-1* upregulation upon LD administration, the use of AA could sustain the anabolic activity of tenocytes during collagen synthesis and secretion.

These in vitro results suggest that a combined treatment with LD and AA could have a synergistic effect, inducing analgesia without affecting the tenocyte biosynthetic activity based on collagen secretion and maturation and avoiding deleterious effects on cell viability. Conversely, AA was not effective in preventing the pro-oxidant effect of LD and in normalizing MMP-1 levels, suggesting that oxidative stress and excessive collagen degradation could be detrimental in LD-treated tendons. However, LD used at the 2% clinically referenced dose is easily adsorbed from the injection site due to its chemical structure [63], reducing the risk of tendon injury. LD’s rapid clearance from the joint cavity to the systemic circulation was demonstrated in horses, showing a 33–61% concentration reduction 7 min after the injection, and further confirmed in dogs, in which the clearance of LD from the joint into serum already started 5 min after intra-articular injection [25,64,65].

## 5. Conclusions

Considered as a whole, our in vitro findings contribute to clarifying how LD potentially impacts on tenocytes and tendon ECM homeostasis. Although the effect of LD on elastin and proteoglycans was not investigated, for the first time, to our knowledge, all the fundamental steps of synthesis, maturation, secretion, and degradation that are part of the collagen turnover process were analyzed. Moreover—though with the limitations of an in vitro model—our results provide new additional information that can be useful for the safe clinical application of these drugs for peritrochanteric infiltrations, suggesting that LD is not detrimental in terms of tenocyte viability. Indeed, the use of LD can offer relevant main advantages such as immediate pain relief, reducing procedural discomfort and helping the physician to confirm that they have reached the target zone; furthermore, it could be useful as a diagnostic test to confirm the origin of the pain. Finally, considering its documented anti-inflammatory properties mediated by the reduction in cytokine release, LD injection could be beneficial for the modulation of the inflammatory processes [13,66,67], thus supporting its use in the treatment of trochanteric bursitis, the predominant cause of GTPS [68]. However, although the anti-inflammatory mechanism of lidocaine is not fully understood, further studies are needed to characterize LD’s anti-inflammatory effect in cultured tenocytes.

## Data Availability

The original contributions presented in this study are included in the article. Further inquiries can be directed to the corresponding authors.

## Abbreviations

The following abbreviations are used in this manuscript:

AA	Ascorbic acid
DCFDA	2′,7′-dichlorofluorescin diacetate
ECM	Extracellular matrix
COL-I	Collagen type I
GTPS	Greater trochanteric pain syndrome
HO-1	Heme oxygenase 1
LA	Local anesthetic
LD	Lidocaine
LH2b	Long lysyl hydroxylase 2
LOX	Lysyl oxidase
MMP	Matrix metalloproteinase
ROS	Reactive oxygen species
TIMP	Tissue inhibitor of matrix metalloproteinase
